# Quantitating skill acquisition with optical ultrasound simulation

**DOI:** 10.1002/ajum.12221

**Published:** 2020-08-02

**Authors:** Anna E Clark, Caroline J Shaw, Fernando Bello, Gihad E Chalouhi, Christoph C Lees

**Affiliations:** ^1^ Queen Charlotte’s and Chelsea Hospital Imperial Healthcare NHS Trust London UK; ^2^ Institute of Reproductive and Developmental Biology, Department of Metabolism, Digestion and Reproduction Imperial College London London UK; ^3^ Faculty of Medicine, Department of Surgery & Cancer Imperial College London Chelsea and Westminster Campus London UK; ^4^ École de Simulation pour L'enseignement et le Perfectionnement en Échographie Gynécologique et Obstétricale (SimECHOle) Paris France; ^5^ Department of Obstetrics and Gynecology Division of Fetal Medicine American University of Beirut Medical Center American University of Beirut Beirut Lebanon; ^6^ Basic Training Task Force Education Committee International Society of Ultrasound in Obstetrics and Gynecology (ISUOG) London UK; ^7^ Department of Development and Regeneration KU Leuven B ‐ 3000 Leuven Belgium

**Keywords:** education, ultrasound simulation, ultrasound training

## Abstract

**Objective:**

To investigate and compare the effect of simulator training on quantitative scores for ultrasound‐related skills for trainees with novice level ultrasound experience and expert ultrasound operators.

**Methods:**

Three novice (comprising of 11, 32, 23 participants) and one expert (10 participants) subgroups undertook an ultrasound simulation training session. Pre‐ and post‐training test scores were collected for each subgroup. Outcome measures were as follows: mean accuracy score for obtaining the correct anatomical plane, percentage of correctly acquired target planes, mean number of movements, time to achieve image, distance travelled by probe and accumulated angling of the probe.

**Results:**

The novices showed improvement in image acquisition after completion of the simulation training session with an improvement in the rate of correctly acquired target planes from 28–57% to 39–83%. This was not replicated in the experts. The novice’s individual ratios based on pre‐ vs. post‐training metrics improved between 1.7‐ and 4.3‐fold for number of movements, 1.9‐ and 6.7‐fold for distance, 2.0‐ and 5.2‐fold for time taken and 1.8‐ and 7.3‐fold for accumulated angling. Among the experts, there was no relationship between pre‐training simulator metrics and years of ultrasound experience.

**Conclusions:**

The individual simulation metrics suggest the sessions were delivered at an appropriate level for basic training as novice trainees were able to show demonstrable improvements in both efficiency and accuracy on the simulator. Experts did not improve after the simulation modules, and the novice scores post‐training were similar to those of experts, suggesting the exercises were valid in testing ultrasound skills at novice but not expert level.

## Introduction

The quality of information generated by any diagnostic imaging modality is dependent on both operator skill and clinical experience. This is particularly the case with ultrasound examinations. For many trainee doctors in obstetrics and gynaecology (O&G), the acquisition of practical ultrasound skills is an unstructured process, heavily reliant on supervised ‘hands‐on training’, which can be of variable quality. Unlike structured sonography training, experience is often opportunistic, with the potential for inconsistency in technique, knowledge and range of cases experienced.^1^

In the UK, the Royal College of Obstetricians and Gynaecologists (RCOG) has a competency‐based ultrasound training programme, rather than one based on a minimum number of scans, in which trainees are required to meet a ‘basic’ level of proficiency in early pregnancy and obstetric ultrasound examinations.^2^ Despite this, training opportunities remain limited: a survey of UK East Midland O&G trainee doctors found that only 7% received dedicated ultrasound training on a weekly basis, with 69% rarely having allocated time for ultrasound training.^1^

Recommendations regarding the minimum training requirements to perform O&G ultrasound examinations independently vary widely. The International Society of Ultrasound in Obstetrics and Gynaecology (ISUOG) recommends a minimum of 100 h of supervised scanning, to include 100 obstetric scans and 100 gynaecological scans in order to achieve competency in ‘basic’ ultrasound skills.^3^ The European Federation of Societies for Ultrasound in Medicine and Biology (EFSUMB) recommend a minimum of 500 supervised examinations should be performed over 3–4 months.^4^. However, in a study in which ultrasound‐derived estimated fetal weight (EFW) was compared with actual birthweights (BW), a minimum of 24‐month ultrasound training was required to ensure that more than 70% of EFWs were within 10% of BW.^5^ Despite this, a recent study showed that, while there is an association between number of scans performed and diagnostic accuracy, the number of scans performed is not a sufficiently robust predictor of accuracy to ensure proficiency.^6^

As a result of both the time and subjectivity involved in developing ultrasound skills, there is evolving interest in the use of ultrasound simulation training to enable practical skills to be developed at a time and place convenient to trainee and trainer and as an objective and standardised assessment of competency.^6^, ^7^, ^8^ Several platforms and systems have been described and are reported to lead to sustained improvements in performance, which can be reproduced in the clinical environment.^7^, ^8^, ^9^, ^10^, ^11^, ^12^

### Aims

In this study, we use the in‐built simulator metrics of an ultrasound simulation system to investigate the effect of simulator training on the quality and efficiency of ultrasound images obtained by trainee doctors and sonographers with minimal ultrasound experience. We then investigated the performance of expert ultrasound practitioners on the simulator and compared that with the trainee’s performance.

## Methods

Data were collected between December 2017 and August 2019. The obstetric simulation data were collected in London, UK, and the gynaecology simulation data in Rotterdam, the Netherlands. Ethical approval was not required as this study did not fulfil the HRA requirements for research ethics consideration.

### Participants

Four subgroups were included in this study, each of which completed different training modules, and had different levels of prior experience. (i) Sonography and O&G trainee doctors, novice in performing ultrasound (novices, n = 11, UK), completed the advanced orientation training module in a 90‐min structured ultrasound training session in a classroom‐based setting. (ii) Gynaecology trainee doctors (novices, n = 32, the Netherlands) completed the gynaecology training module in a 2‐h structured ultrasound training session in a classroom‐based setting. (iii) O&G trainee doctors (novices, n = 23, UK) completed a 3‐h structured ultrasound training session during which they completed basic and advanced orientation training modules and the second‐trimester training module in a classroom‐based setting. (iv) The expert group (qualified sonographers or fetal medicine specialists, prior experience of 2‐29 years, n = 10, UK) completed the basic orientation, advanced orientation and second‐trimester training modules in an unstructured workplace‐based session. Participants were allowed up to 3 h to complete all modules. This group comprised three fetal medicine consultants, two subspecialty fetal–maternal medicine trainees and five qualified sonographers.

### Optical ultrasound simulator

**Figure 1:**
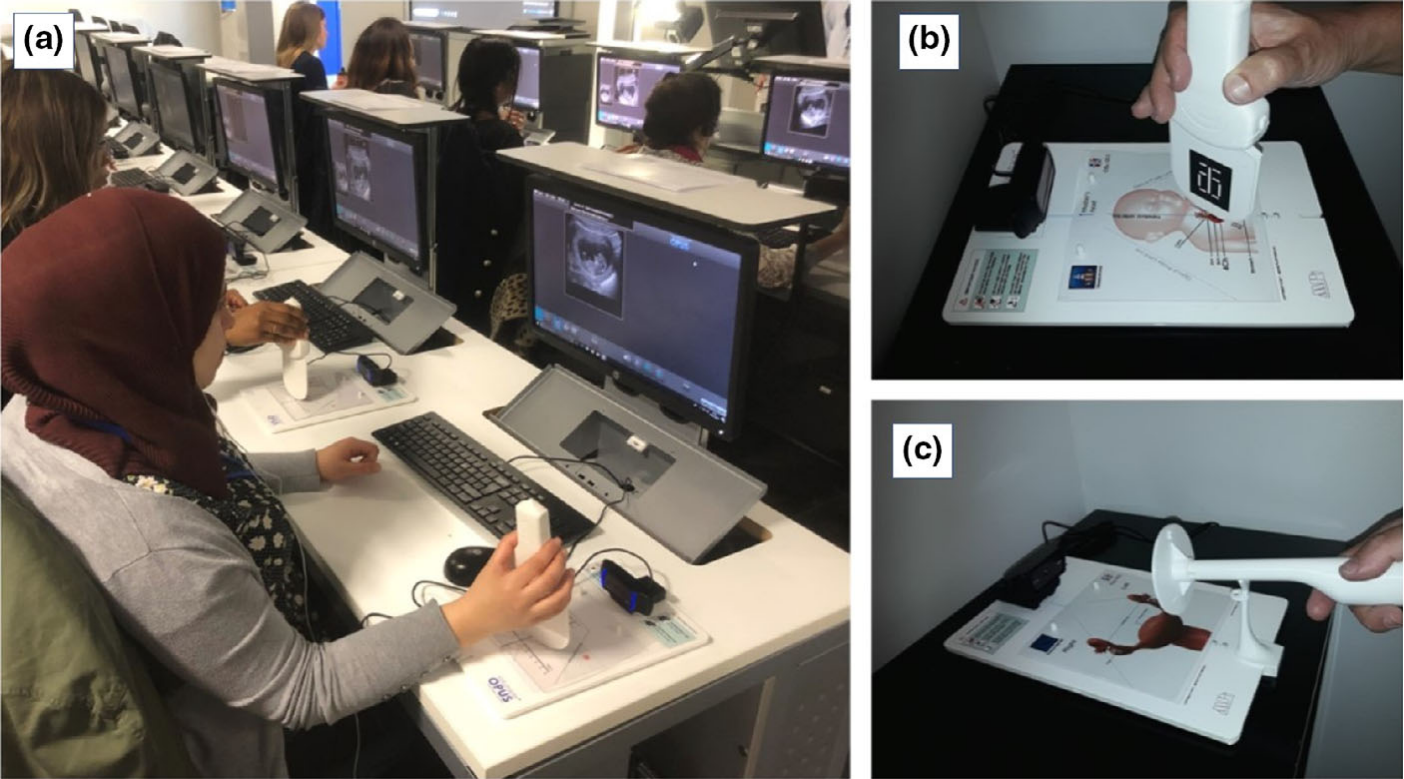
Volutracer OPUS^®^ system (Medge Platforms Inc), (a) represents optimal set‐up. (b) shows the Opus simulator with transabdominal probe. (c) shows the OPUS simulator with transvaginal probe. [Colour figure can be viewed at wileyonlinelibrary.com]

The Volutracer OPUS^®^ system (Medge Platforms Inc, New York City, NY, USA)^13^ is a compact optical simulator, with fully automated web‐based simulation metrics, capable of delivering ultrasound training mapped to meet the basic proficiency requirements of the RCOG ultrasound curriculum. It consists of a plastic replica ultrasound probe (transabdominal/transvaginal) used with a flat scanning pad and camera, which creates 2D ultrasound images on the user’s PC/laptop (Figure 1).

The simulator uses anatomical volumes derived from real patients and optical positioning technology to show the user a 2D ultrasound image relative to the position of the replica probe. The in‐built simulator metrics evaluate transducer movements by measuring each movement of the probe in its six degrees of freedom. This allows the system to measure how accurate the user is by assessing how close to the target plane the image generated by the user is and determining an accuracy score. During tutorials, the simulator guides the student (by instructing movements in any of the six degrees of freedom) to reach the target planes accurately, thereby reducing the need for one‐on‐one teaching.

The accuracy score is calculated by dividing the target plane into 6 different parameters (the 6 degrees of freedom of the ultrasound probe). A distance limit is set for each direction of movement away from the target plane, and this ‘acceptable limit’ is given the score of 7/10. Distances closer to the target plane will score higher, while further away will score progressively lower. Once the trainee is satisfied with the image they have obtained, the simulator allocates an overall accuracy score, which is equal to the lowest scoring parameter. The proportion of images scoring ≥7/10 was also recorded. The system measures the ‘fluency’ of user movements by tracking the time taken to obtain a target plane, the distance travelled, the accumulated angling of the probe’s path and the number of movements of the probe made during this time. During the simulation session, these data are recorded and available for offline analysis thereafter.

### Ultrasound simulator training modules

The participants were all given a demonstration of simulator operation and completed a preparatory module to familiarise themselves with the interface and functional methods of the simulation system before commencing the training session. A faculty member or researcher was present throughout to provide technical support if required.

#### First trimester – basic

This training module presented an anatomical volume of an 11‐week gestation fetus. A transabdominal replica probed was used. Target images were as follows: (i) crown–rump–length view, (ii) positioning the fetal head on a target line in the centre of the monitor and (iii) positioning the fetal limbs on a target line. Participants were asked to complete a pre‐ and post‐training test. Where pre‐ and post‐training scores were not available, participants were excluded from analysis.

#### First trimester – advanced

This training module presented an anatomical volume of an 11‐week gestation fetus. A transabdominal replica probed was used. Target images were as follows: (i) sagittal plane of the head, (ii) positioning the nasal bone on a target line in the centre of the monitor and (iii) positioning the umbilical cord insertion on a target line. Participants were asked to complete a pre‐ and post‐training test. Where pre‐ and post‐training scores were not available, participants were excluded from analysis.

#### Second trimester

This training module presented an anatomical volume of a 20‐week gestation fetus. A transabdominal replica probe was used. The target images were (i) identification of the stomach, (ii) identification of the bladder, (iii) identification of the femur and (iv) identification of genitalia. Pre‐ and post‐training scores were calculated using the metric scores for the first and last image obtained of each target plane. In the cases where the target plane was not repeated more than once (first image already adequate), the metric scores for that target plane were used as both the pre‐ and post‐training result.

#### Gynaecological module

This training module presented an anatomical volume of the female pelvis. A transvaginal replica probe was used. The target images were as follows: (i) longitudinal view of the uterus – two targets, (ii) transverse view of the uterus, (iii) longitudinal view of the embryo, (iv) transverse view of the embryo, (v) longitudinal view of the left ovary, (vi) longitudinal view of the right ovary, (vii) transverse view of the left ovary, (viii) transverse view of the right ovary, (ix) transverse view of the cervix, (x) transverse view of fundus of uterus and (xi) location of intrauterine device. Participants were asked to complete a pre‐ and post‐training test. Where pre‐ and post‐training scores were not available, participants were excluded from analysis. Up to 13 users did not provide data for analysis of both the pre‐ and post‐training tests. Pre‐test user numbers: image 1‐4 n = 32, image 5 n = 31, image 6‐10 n = 30, image 11 n = 29. Post‐test user numbers: image 1‐8 n = 21, image 9‐10 n = 20, image 11 n = 19.

### Statistical analysis

Statistical analysis was performed in SPSS 22.0 (SPSS Inc., Chicago, IL, USA). Statistical significance was accepted when P < 0.05. Where applicable, individual P‐values are presented in tables and text.

Continuous data were assessed for normality with the Shapiro–Wilk test. Descriptive analysis was performed using mean ± standard deviation (SD), unless otherwise stated. Ratios of individual improvement were calculated by dividing the pre‐training score by the post‐training score for number of movements, time, distance moved and accumulated angulation to achieve an adequate image.

For normally distributed data, means were compared with two‐tailed Student’s *t‐*test. To assess the effect of simulation training on a continuous variable (simulator metrics), when the independent variable of previous experience needed to be considered, a two‐way ANOVA was used to investigate the change in simulator metric scores due to the interaction of previous experience of ultrasound and completion of simulator training. If a significant effect or interaction was identified, post hoc testing was performed as applicable to identify the source of the variation.

Correlation between expert practitioners’ years of experience and optical simulator metric scores was assessed using the Spearman rank correlation coefficient.

## Results

All three novice groups showed improvement following training on the optical simulator, whereas those in the expert group did not.

Among the novices in subgroup 1, following completion of the first‐trimester advanced module, there was a higher accuracy score of 6.4 ± 0.4 compared to 5.7 ± 0.9 prior to the training (P = 0.02). Similarly, there was improvement in the rate at which novices correctly acquired target planes and a reduction in the number of movements, time to acquire an image, total distance travelled by the probe and in the accumulated probe angulation (Table 1).

**Table 1:** Pre‐ and post‐training test scores.

Session	Module	Metric	Pre‐test	Post‐test	P value
1. Novices (O&G and sonographer trainees)	Obstetric simulation First trimester advanced	Accuracy score (maximum 10)	5.7 ± 0.9	6.4 ± 0.4	0.02^*^
		Rate of correctly acquired target planes (%)	38% ± 13	47% ± 12	0.04^*^
		No. of movements	34 ± 16	21 ± 9	0.004^*^
		Time (s)	26 ± 11	17 ± 6	0.006^*^
		Distance (cm)	18 ± 12	8 ± 4	0.02^*^
		Accumulated angling (degree)	236 ± 157	141 ± 62	0.04^*^
2. Novices	Gynaecology simulation	Accuracy score (maximum 10)	4.0 ± 2.9	5.1 ± 2.6	<0.001^*^
		Rate of correctly acquired target planes (%)	28% ± 45	39% ± 49	0.004^*^
		No. of movements	23 ± 34	12 ± 11	<0.001^*^
		Time (s)	22 ± 33	12 ± 11	<0.001^*^
		Distance (cm)	25 ± 52	12 ± 25	<0.001^*^
		Accumulated angling (degree)	316 ± 756	138 ± 327	<0.001^*^
3. Novices (O&G trainees)	Obstetric simulation First trimester basic	Accuracy score (maximum 10)	6.5 ± 0.6	7 ± 0.2	0.03^*^
		Rate of correctly acquired target planes (%)	57% ± 18	69% ± 14	0.03^*^
		No. of movements	46 ± 17	23 ± 9	<0.001^*^
		Time (s)	56 ± 23	24 ± 8	<0.001^*^
		Distance (cm)	33 ± 25	14 ± 8	<0.001^*^
		Accumulated angling (degree)	465 ± 297	265 ± 174	0.004^*^
	Obstetric simulation First trimester advanced	Accuracy score (maximum 10)	6.4 ± 0.7	6.7 ± 0.5	0.09
		Rate of correctly acquired target planes (%)	51% ± 16	57% ± 17	0.39
		No. of movements	32 ± 11	21 ± 9	<0.001^*^
		Time (s)	40 ± 14	21 ± 8	0.28
		Distance (cm)	19 ± 7	14 ± 11	0.13
		Accumulated angling (degree)	285 ± 115	208 ± 156	0.19
	Second trimester	Accuracy score (maximum 10)	5.5 ± 1	6.5 ± 1	<0.001^*^
		Rate of correctly acquired target planes (%)	45% ± 27	83% ± 20	<0.001^*^
		No. of movements	59 ± 39	32 ± 22	0.001^*^
		Time (s)	93 ± 78	44 ± 41	0.003^*^
		Distance (cm)	50 ± 46	23 ± 24	0.005^*^
		Accumulated angling (degree)	611 ± 583	277 ± 332	0.005^*^
4. Experts	Obstetric simulation First trimester basic	Accuracy score (maximum 10)	6.8 ± 0.6	7.2 ± 0.3	0.19
		Rate of correctly acquired target planes (%)	66% ± 11	65% ± 8	>0.99
		No. of movements	20 ± 9	18 ± 7	0.83
		Time (s)	27 ± 9	23 ± 16	0.77
		Distance (cm)	13 ± 5	11 ± 8	0.93
		Accumulated angling (degree)	186 ± 93	164 ± 127	0.94
	Obstetric simulation First trimester advanced	Accuracy score (maximum 10)	6.6 ± 0.4	6.9 ± 0.4	0.23
		Rate of correctly acquired target planes (%)	62% ± 8	61% ± 8	0.99
		No. of movements	21 ± 6	18 ± 9	0.54
		Time (s)	25 ± 8	46 ± 69	0.29
		Distance (cm)	13 ± 7	10 ± 8	0.61
		Accumulated angling (degree)	192 ± 105	149 ± 121	0.66
	Second trimester	Accuracy score (maximum 10)	6.8 ± 0.8	7.4 ± 0.3	0.13
		Rate of correctly acquired target planes (%)	68% ± 27	100% ± 0	0.005^*^
		No. of movements	14 ± 12	12 ± 13	0.99
		Time (s)	20 ± 15	18 ± 16	0.99
		Distance (cm)	9 ± 7	8 ± 8	>0.99
		Accumulated angling (degree)	68 ± 58	63 ± 65	>0.99

Values represent mean ± standard deviation (SD) of the accuracy score, rate of adequate acquisition of target planes, the number of probe movements, the time taken, the cumulative distance the probe was moved and the accumulated angulation of the probe to achieve an ultrasound image the user felt was adequate in pre‐ and post‐training module tests.

* *P* values are Statistically significant.

The novices (subgroup 3) improved their accuracy score from 6.5 ± 0.6 to 7.0 ± 0.2 (P = 0.03) following completion of the first‐trimester basic module, while improving the rate of correctly acquired target planes and reducing the number of movements, time, distance and angulation required to achieve an adequate image. However, there was no corresponding improvement in the accuracy score following completion of the first‐trimester advanced module with a pre‐module score of 6.4 ± 0.7 and a post‐module score of 6.7 ± 0.5 (P = 0.09). The only improvements noted in this subgroup resulting from completion of the first‐trimester advanced module were in the number of movements. These novices also improved their accuracy score from 5.5 ± 1 to 6.5 ± 1 (P = <0.001) following completion of the second‐trimester module, with corresponding improvements in rate of correctly acquired target planes, and reduction in number of movements, time, distance and angulation required to achieve the correct plane (Table 1).

The novices completing the gynaecology simulation module also improved their accuracy scores from 4.0 ± 2.9 to 5.1 ± 2.6 (P = <0.001) with corresponding improvements in rate of correctly acquired target planes, and reduction in number of movements, time, distance and angulation required to achieve the correct plane (Table 1).

The expert group had a pre‐test accuracy score of 6.8 ± 0.6 in the first‐trimester basic module, 6.6 ± 0.4 in the first‐trimester advanced module and 6.8 ± 0.8 in the second‐trimester module. They showed no improvement in accuracy score, number of movements, time, distance or angulation of the probe required to achieve the correct plane following completion of any of the three training modules (Table 1). There was no correlation between the years of experience of a member of the expert group and any pre‐training simulator metric: accuracy score (*r* ^2^ = −0.4080, P = 0.24), percentage of correctly acquired target planes (*r* ^2^ = −0.41, P = 0.23), movements (*r* ^2^ = 0.02, P = 0.95), time (*r* ^2^ = −0.08, P = 0.83), distance (*r* ^2^ = 0.01, P = 0.98) and accumulated angling (*r* ^2^ = −0.006, P = 0.99).

Compared to the trainee doctors classified as novices (subgroup 3), the expert group did not have a higher pre‐training accuracy score, or rate of correctly acquired target planes, in either the basic or advanced modules. However, in the first‐trimester basic module, the expert group obtained adequate images quicker, with fewer movements, a shorter distance of probe travel and less accumulated angulation. In the first‐trimester advanced module, the expert group obtained the adequate image with fewer movements than the novices, but otherwise performed similarly to the novices. Following the completion of the basic and advanced training modules, the performance of the novices and the experts was not different as assessed by the metrics of the optical ultrasound simulator.

In the second‐trimester module, the expert group performed better than the novices prior to the training module, with a pre‐test score of 6.8 ± 0.8 compared to 5.5 ± 1.2 (P = 0.002) and a higher rate of acquiring adequate imaging planes, and were quicker to do so using less movements, less distance of travel of the probe and less angulation. By the completion of the training module, the accuracy score of the expert group remained higher at 7.4 ± 0.3 compared to 6.5 ± 1.0 (P = 0.04), but both groups performed similarly in the other metrics (Table 2).

**Table 2:** Novice vs. expert pre‐ and post‐training test scores.

Module	Pre/Post	Metric	Novice	Expert	P value
1. First trimester – basic	Pre	Accuracy score (maximum 10)	6.5 ± 0.6	6.8 ± 0.6	0.34
		Rate of adequate acquisition of target planes (%)	57% ± 18	66% ± 11	0.21
		No. of movements	46 ± 17	20 ± 9	<0.001^*^
		Time (s)	56 ± 23	27 ± 9	<0.001^*^
		Distance (cm)	33 ± 25	13 ± 5	0.003^*^
		Accumulated angling (degree)	465 ± 297	186 ± 93	0.003^*^
	Post	Accuracy score (maximum 10)	7 ± 0.2	7.2 ± 0.3	0.58
		Rate of adequate acquisition of target planes (%)	69% ± 14	65% ± 8	0.77
		No. of movements	23 ± 9	18 ± 7	0.48
		Time (s)	24 ± 8	23 ± 16	0.98
		Distance (cm)	14 ± 8	11 ± 8	0.81
		Accumulated angling (degree)	264 ± 174	164 ± 127	0.4
2. First trimester – advanced	Pre	Accuracy score (maximum 10)	6.4 ± 0.7	6.6 ± 0.4	0.48
		Rate of adequate acquisition of target planes (%)	51% ± 16	62% ± 8	0.12
		No. of movements	32 ± 11	21 ± 6	0.006*
		Time (s)	40 ± 14	25 ± 8	0.5
		Distance (cm)	19 ± 7	13 ± 7	0.25
		Accumulated angling (degree)	285 ± 115	192 ± 105	0.17
	Post	Accuracy score (maximum 10)	6.7 ± 0.5	6.9 ± 0.4	0.58
		Rate of adequate acquisition of target planes (%)	57% ± 17	61% ± 8	0.69
		No. of movements	21 ± 9	18 ± 9	0.63
		Time (s)	21 ± 8	46 ± 69	0.16
		Distance (cm)	14 ± 11	10 ± 8	0.6
		Accumulated angling (degree)	208 ± 156	149 ± 121	0.48
3. Second trimester	Pre	Accuracy score (maximum 10)	5.5 ± 1.2	6.8 ± 0.8	0.002^*^
		Rate of adequate acquisition of target planes (%)	45% ± 27	68% ± 27	0.02^*^
		No. of movements	59 ± 39	14 ± 12	<0.001^*^
		Time (s)	93 ± 78	20 ± 15	0.001^*^
		Distance (cm)	50 ± 46	9 ± 7	0.002^*^
		Accumulated angling (degree)	611 ± 583	68 ± 58	0.001^*^
	Post	Accuracy score (maximum 10)	6.5 ± 1.0	7.4 ± 0.3	0.04^*^
		Rate of adequate acquisition of target planes (%)	83% ± 20	100% ± 0	0.09
		No. of movements	32 ± 22	12 ± 13	0.1182
		Time (s)	44 ± 41	18 ± 16	0.3539
		Distance (cm)	23 ± 24	8 ± 8	0.3720
		Accumulated angling (degree)	277 ± 332	63 ± 65	0.2996

Values represent mean ± standard deviation (SD) of the accuracy score, rate of adequate acquisition of target planes, the number of probe movements, the time taken, the cumulative distance the probe was moved and the accumulated angulation of the probe to achieve an ultrasound image the user felt was adequate in pre‐ and post‐training module tests. Statistically significant p values are denoted by *.

Within the novice group, individual ratios of pre‐ and post‐training metrics for number of movements, time taken, distance moved and accumulated angulation required to achieve an adequate image improved between 2.0‐ and 2.5‐fold in the first‐trimester basic module, 1.7‐ and 2.0‐fold in following completion of the first‐trimester advanced module and 4.3‐ and 7.3‐fold after completion of the second‐trimester module (Table 3).

**Table 3:** Ratio Improvement in novice skills (subgroup 3).

	Time (s)	Distance (cm)	Accumulated angling (degree)	No. of movements
First trimester – basic	2.3 ± 1.2	2.5 ± 1.9	2.0 ± 1.5	2.2 ± 0.9
First trimester – advanced	2.0 ± 0.8	1.9 ± 0.9	1.8 ± 1.0	1.7 ± 0.6
Second trimester	5.2 ± 3.3	6.7 ± 6.8	7.3 ± 4.5	4.3 ± 2.6

Values represent mean ± standard deviation (SD) of ratio improvement for the time taken, the cumulative distance the probe was moved and the accumulated angulation of the probe to achieve an ultrasound image the user felt was adequate in the first‐trimester basic, first‐trimester advanced and second‐trimester training modules.

## Discussion

This study shows that an optical ultrasound simulation system with in‐built metrics can be used to improve the quality, speed, efficiency and accuracy of simulated ultrasound image acquisition in trainee doctors with minimal previous experience of ultrasound scanning. These improvements were achieved during facilitated, structured training sessions of between 90 and 180 min in a classroom‐based setting, timed to the convenience of the trainee and facilitator and demonstrated objectively. Improvements in the post‐training scores in relation to overall score, percentage of correctly acquired target plane, as well as reduction in distance travelled by the probe, accumulated angling, number of movements and time taken to achieve the final image indicate that the novices had become more accurate and proficient in image acquisition on the simulator following completion of these training modules.

Notably, similar improvement was not seen in the expert group, suggesting that the improvements in all metrics in the novice group were not based on increased familiarity with the simulator, rather on improvement in the cognitive and motor skills required to acquire an adequate image, which were already well developed in the experts.

This is supported by the fact that the pre‐ and post‐training scores for the first‐trimester advanced module improved in the subgroup 1 novices (who had not been previously exposed to the first‐trimester basic module), but not in the subgroup 3 novices (who had previously completed the first‐trimester basic module). This suggests that the subgroup 3 novices developed their cognitive and motor skills related to an 11‐week fetus during the basic module and that the advanced module was sufficiently similar to allow transfer of these skills, an advantage the subgroup 1 novices did not have. In fact, the subgroup 1 post‐training score (6.4 ± 0.4) in the first‐trimester advanced module is not higher than the pre‐training score in the subgroup 3 novices for the same module (6.4 ± 0.7). However, despite their prior exposure to the anatomical volume of the 11‐week fetus in the basic module, subgroup 3 novices continued to make reductions in the number of movements required to obtain an adequate image, showing that further training improved the efficiency with which they performed ultrasound examinations.

Comparison of the expert and novice scores in the completion of the first‐trimester basic, advanced and second‐trimester training modules demonstrates the pattern of transfer of skills. In the first‐trimester basic module, the experts scored higher in the pre‐training test than the novices; by the post‐training test, these differences had been negated. In the first‐trimester advanced module completed next, the skills from the basic module were transferred such that there were no differences found between novices and experts. However, the third training module in the sequence, the second‐trimester training module, again showed that the experts performed better than the novices in the pre‐training test. Hence, while the novices had gained familiarity with the 11‐week fetus anatomical volume, these skills were not immediately transferable to a new, 20‐week fetus anatomical volume. This pattern demonstrates that the novices had not reached an expert level of scanning as a result of the training modules; they remained slower to adapt to new anatomical volumes than the expert group. This underlines the importance of introducing varied experience into simulator training: just as hands‐on training can leave gaps in trainee experience, so can a lack of diversity in anatomical volumes provided for simulator training. Simulator training content and anatomical volumes should hence ideally be mapped to a sequential curriculum. While assessment of competency is likely to continue to be assessed in the real‐world setting, our results demonstrate that novices can develop transferable ultrasound skills that may benefit them.

As a crude measure of improvement, when the ratio of pre‐ and post‐training scores is considered, the difference between the first‐trimester basic and advanced modules and the second‐trimester training module is pronounced. The second‐trimester training module required a wider variety of techniques to image the anatomical volume in sagittal, coronal and axial planes, compared to the basic and advanced orientation module, which required imaging in the sagittal plane only. Greater ratios of improvement were seen in novices completing the second‐trimester training module than in the first‐trimester basic and advanced modules. However, we cannot determine whether this is because the second‐trimester training module develops skills more effectively, or whether it was simply more challenging than the first‐trimester basic and advanced modules.

It is important to note that there is limited evidence regarding the validity of simulator metrics. The scoring of ultrasound skill acquisition is traditionally manual and subjective, with external assessors using preformatted scoring templates to reduce variability as far as possible. The use of automatic simulator metrics may offer a solution. However, previous studies have demonstrated that only one‐third of in‐built metrics are able to reliably differentiate between novice and expert level.^14^, ^15^ The difference between these and our study is that, rather than examining the validity of a pass/fail level as a potential determinant of competence, we have looked at different measures of performance metrics to determine whether there are differing characteristics between novice and experts performance on this simulator and whether these metrics have the potential to be used as a measure of skill acquisition related to ultrasound training.

This study has some limitations. The participant numbers in each group were relatively modest, though sufficient to demonstrate differences in the specific skills measured. We had expected that there would be a linear relationship between the expert’s years of experience and their simulator scores, which was not found in this study. The reason for this may be threefold. Firstly, the modules and target planes we selected were designed to be accessible to novices and may have been insufficiently challenging to elucidate differences between skill levels in our expert group, who could all perform basic skills competently. Secondly, while the expert group acquired images more efficiently than the novice group, their mean accuracy scores remained around the 7.0/10 level – the minimum level the system accepted to allow a user to progress to the next stage. Therefore, the system was accepting of a degree of error, which may not have ‘pushed’ experts to achieve the quality of image they were capable of, especially given that their sessions were unstructured and took place during the normal workday. Thirdly, the metrics used may be suitable to determine basic competence but may not be able to determine more subtle gradations in skill level beyond this.

In order for simulation training to be a valuable training resource, the skills and theoretical knowledge learned during simulation training must be transferrable to the clinical setting and challenges therein. Performing a complete obstetric ultrasound examination requires a combination of cognitive skills (working memory to plan and conduct the examination and image interpretation,) fine motor skills, communication skills, professional judgement, obstetric knowledge and clinical decision making. Simulation training is focused on developing cognitive and fine motor skills and reinforcing theoretical knowledge. Developing the clinical acumen to interpret, manage and communicate a range of scan findings can only be learnt by the bedside with real patients over time. As such simulation cannot replace training in real‐life clinical scenarios, but does give a chance to practice skills known to improve with repetition in a controlled environment.^16^ We anticipate that this skill baseline would assist trainees to respond to challenges such as oligohydramnios, increased BMI or poor fetal position. However, current data to support this are not available and would be a valuable avenue of further investigation.

In summary, achieving ultrasound competency can be challenging for trainees. Practical ‘hands‐on’ training on real patients with a supervising ultrasound expert is the gold standard but requires time and staffing. Both these commodities are in short supply in many healthcare systems throughout the world. Moreover, using real‐time patients for teaching transvaginal ultrasound has ethical considerations.^17^, ^18^ The authors believe, if trainees had regular access to a personal, compact, optical ultrasound simulation system allowing fully objective analysis of several parameters in addition to simulation modules that were appropriate to their level of training and clinical duties, then the use of ultrasound simulation alongside clinical training would act as a useful adjunct and bridge the gap between theory and clinical practice. It would also allow multiple students to undertake training simultaneously, each with their own simulator for both transvaginal and transabdominal ultrasound. Assessing how well skills gained on ultrasound simulation subsequently translate to the clinical environment remains unelucidated and further work is warranted to determine how best to optimise the simulation curriculum and module content to best facilitate and assess this.

## Authorship statement

CCL, GEC, AEC and FB conceived and designed the study; AEC, GEC and FB were involved in data collection; AEC and CJS analysed the data for publication. AEC, CJS, FB, GEC and CCL drafted the article and revised it for important intellectual content.

## Funding

No funding information is provided.

## Conflict of interest

The authors declare no competing interests, financial or otherwise.
